# Inflammation and quality of life in later life: findings from the health, well-being and aging study (SABE)

**DOI:** 10.1186/s12955-019-1092-2

**Published:** 2019-02-06

**Authors:** Manuela de Almeida Roediger, Maria de Fátima Nunes Marucci, Etienne Larissa Duim, Jair Lício Ferreira Santos, Yeda Aparecida de Oliveira Duarte, Cesar de Oliveira

**Affiliations:** 1 School of Public Health, University of São Paulo, São Paulo, Brazil; 2 Department of Nutrition, School of Public Health, University of São Paulo, São Paulo, Brazil; 3 Department of Social Medicine, University of São Paulo, Ribeirão Preto, Brazil; 4 Department of Nursing Medical Surgical, University of São Paulo, São Paulo, Brazil; 5 Department of Epidemiology & Public Health, University College London, London, UK; 6 Department of Nutrition, Faculty of Public Health (FSP), University of Sao Paulo (USP), Avenida Doutor Arnaldo 715, Cerqueira César, São Paulo - SP CEP: 01246-904 Brazil

**Keywords:** Inflammation, Quality of life, Aging, SABE study, Brazil

## Abstract

**Background:**

Few studies have specifically investigated the inverse relationship between reduced quality of life in different domains and elevated C-reactive protein (CRP) serum levels in older adults. Therefore, this study investigates the cross-sectional association between quality of life and inflammation in older Brazilian adults.

**Methods:**

Data were collected from 1255 participants from the third wave (2010) of the Brazilian Health, Well-being and Aging study (SABE), a community-based cohort study of aging. Inflammation was assessed using CRP serum levels and quality of life (QoL) was measured using the 12-item Short-Form Health Survey (SF-12) questionnaire. The covariates included age, sex, education level, financial sufficiency, number of non-communicable diseases, self-reported doctor diagnosed diseases, Activity of Daily Living (ADL) difficulties, Body Mass Index (BMI), and waist circumference.

**Results:**

The fully adjusted models showed that older adults with low scores in the physical domain of the SF12 (OR 1.34, 95%CI 1.02;1.77) and high BMI values (> 30) (OR 2.05, 95%CI 1.50;2.81) were more likely to present high CRP serum levels.

**Conclusion:**

Our findings suggest a significant association of lower scores in the physical domain of quality of life and the presence of obesity with high CRP serum levels.

## Introduction

Robust literature already exists on chronic disease and quality of life (QoL) [1–4]. Much of this evidence has focused on chronic inflammation, which may play a role in a wide range of chronic conditions that could negatively affect QoL [5, 6]. In addition, there has been growing interest in medical sociology research using biomarker data to investigate how non-biological factors including emotional, behavioral, affective, physical, and social information may predispose people to inflammation [7–10].

Previous studies have demonstrated a relationship between inflammatory biomarkers such as Interleukin (IL)-6, IL-18, and IL-10, tumor necrosis factor alpha (TNF-alpha), and interferon gamma with chronic diseases, mental disorders, and obesity and overweight, as well as self-rated health [4, 11]. The inflammatory biomarker C-reactive protein (CRP) has also been widely used in socio-medical research. Elevated levels of this biomarker in serum can indicate progression of inflammation in individuals with chronic conditions [6, 12, 13]. CRP is a good predictor of chronic inflammation and its mean value increases with age [4, 6]. Moreover, elevated CRP levels have been shown to be associated with coronary heart disease, cancer, inflammatory diseases, respiratory disorders, chronic kidney disease, bacterial or viral infections, diabetes, obesity, metabolic syndrome, psychiatric problems, depression, and other diseases. In general, CRP is a valuable inflammatory biomarker in various clinical conditions [6, 12–15].

The role of inflammation in the aging process and age-associated chronic conditions has been clearly established in several epidemiologic studies conducted in older adults [4, 5, 8, 16]. Low-grade elevation of inflammatory markers such as CRP in older adults has been associated with a number of chronic conditions, such as cardiovascular disease, diabetes, physical disability, and cognitive decline [4, 16, 17]. In older adults, elevated CRP levels are a common phenomenon and have been associated with lower levels of QoL. Therefore, the association between CRP serum levels and QoL later in life may offer interesting interventional prospects [4, 5, 8].

The relationship between chronic inflammation and quality of life builds on research from social and clinical disciplines connecting chronic conditions and inflammation to reduced quality of life [5, 7, 9, 18]. However, few studies have specifically investigated the inverse relationship between reduced quality of life in different domains and elevated C-reactive protein (CRP) serum levels in older adults [18, 19], as well as whether there are differences between genders.

We hypothesized that lower perceived health related quality of life could be associated with elevated levels of CRP. In a context of an accelerated aging process and considering how inflammation can affect the health of older adults, it is important to understand mechanisms associated with higher levels of inflammation in this population. This study contributes to a growing body of evidence on the relationship between quality of life and inflammation in later life and aims to investigate the association between quality of life and inflammation in older Brazilian adults using data from the Health, Well-being and Aging study (SABE), a community-based cohort study of aging in Brazil.

## Methods

### Study design and population

The Health, Well-being and Aging (SABE) Study is a panel study that began in 2000, and forms part of a multicenter survey carried out in the main urban centers of seven countries in Latin America and the Caribbean. In Brazil, 2143 older adults living in Sao Paulo city were selected through a probabilistic sample, representative of the urban population aged 60 years and older. At baseline, the data collection involved an at-home interview, anthropometric measures, and physical performance tests. After baseline, follow-up interviews within SABE occur every 5 years. A detailed description of the SABE study design and sampling process has been published previously [20]. For this cross-sectional analysis, we used data from 1255 participants who took part in the third wave of the SABE cohort in 2010. Information was collected through a structured questionnaire administered by trained interviewers and blood samples collected at the participant’s home by a qualified health professional [21, 22].

The SABE study was approved by the Ethics in Research Committee of the School of Public Health of the University of Sao Paulo and by the Brazilian National Committee for Ethics in Research [20, 21]. As inclusion Criteria, participants who gave written informed consent to blood tests, and presented complete information on the SF12 questionnaire and all covariates were included in our analyses.

### Assessment of inflammation

Serum aliquots were frozen and stored at − 80 °C until enzyme-linked immunosorbent assay (ELISA) tests were performed. CRP concentrations were measured using an immunoturbidimetric assay (Roche Diagnostics). The results were expressed as milligram per liter. CRP serum levels were analyzed both as a continuous variable, by considering their relationship with age and gender, and dichotomized, classifying participants into two groups: normal CRP level (≤ 3.0 mg/L) and high CRP level (> 3.0 mg/L) [23, 24].

### Quality of life (QoL) assessment

QoL was assessed using the 12-Item Short Form Survey (SF12), a multidimensional instrument validated for health related quality of life [25, 26]. The SF12 contains 12 items that were combined, scored, and weighted to create two scales that provide glimpses into mental and physical functioning and overall health-related-quality of life. Scores range from 0, indicating the lowest QoL, to 100, the highest QoL. In this analysis, we used SF12 tertiles, a statistical procedure adopted due to the non-parametric distribution of this variable. The reference category was the highest tertile [27].

### Covariates

Covariates included the following sociodemographic variables: sex, age groups (60–69; 70–79; 80 years and older), educational level (illiterate, 1–3 years, 4–7 years, 8 and more years of schooling), and financial sufficiency assessed by answers to a direct question: “Do you think you have enough money for your expenses?”. Health conditions were the number of non-communicable diseases (none, one, 2 or more) and self-reported doctor diagnosed chronic conditions (chronic pain, hypertension, diabetes, cardiovascular disease, diabetes, and arthritis). Participants were asked if they had any difficulty performing one or more Activities of Daily Living (ADL) (i.e., walking, transferring, toileting, bathing, dressing, or feeding). The Body Mass Index (BMI) was categorized according to the Pan-American Organization recommendation (undernutrition: BMI < 23.0; Normal ≥23 and < 28.0; overweight ≥28 and < 30, Obesity≥30) [28]. Waist circumference (WC) was categorized according to cardiovascular risk and metabolic diseases (WC > =94 cm for men, and > =80 for women) [29]. Cognitive function was assessed using the Mini Mental State Examination, and individuals who scored 12 points or less were classified with low cognitive function [30].

### Statistical analysis

Descriptive data are expressed as proportions for categorical variables and means and standard errors for continuous variables to describe sociodemographic and health variables. Means and their 95% confidence intervals are presented for CRP serum levels and for the physical and mental domains of the SF12 questionnaire, stratified by sex. The association between inflammation and quality of life was investigated using the Chi-square test with Rao & Scott correction. Univariate and multiple logistic regressions, adjusted by a wide range of covariates, are presented with odds ratio (OR) as a measure of effect size and their respective 95% confidence intervals (CI). Statistical analyses were conducted using Stata® version 13 (Stata Corp, College Station, TX). As the SABE study is based on a probabilistic sample, representative of the urban population aged 60 years and older living in Sao Paulo city, all analyses used the survey mode (i.e. *svy*) to account for the complex sample design and sampling weights.

## Results

The participants’ characteristics are shown in Table 1. Of the 1344 older adults aged 60 and older who took part in the SABE study in 2010, 1255 were eligible and included in our analyses. Overall, there were more participants from the younger age group (i.e., 60–69), with low educational level, and approximately 40% reported that they did not have enough income to pay for their expenses. More than 80% of the sample had one or more non-communicable disease (NCD) and hypertension was the most prevalent self-reported chronic condition. Only 17.5% reported any difficulty in performing ADL. Obesity was observed in 32.4% of the sample, being more prevalent in women. Women also presented lower quality of life scores for the physical and mental domains compared to men.

**Table 1:** Sample characteristics of 1255 participants aged 60 years and older, by gender, from the SABE Study (Health, Well-Being, and Aging), Brazil, 2010

Variables	Male	Female	Total	*P* Value
Gender	40.1	59.9	100	
CRP (95%CI)^a^	5.21 (4.28;6.15)	4.45 (3.94;4.96)	4.76 (4.30;5.12)	–
CRP				
≤ 3.0 mg/L	42.2	57.8	58.7	
> 3.0 mg/L	36.3	63.7	41.3	0.08
Quality of Life Measure				
Physical domain	54.7 (53.6;55.8)	50.7 (49.6;51.8)	52.3 (51.4;53.3)	
Mental domain	61.4 (60.6;62.3)	58.5 (57.4;59.6)	59.7 (58.9;60.5)	–
Age				
60–69	42.8	57.2	54.2	
70–79	39.1	60.9	30.5	
80 and older	32.5	67.5	15.3	0.028
Educational level				
Illiterate	33.1	66.9	11.9	
1–3 years	34.2	65.8	22.5	
4–7 years	40.8	59.2	37.5	
8 and more years	46.6	53.4	28.0	0.005
Financial sufficiency				
Yes	43.1	56.9	57.0	
No	36.2	63.8	43.0	0.029
Number of Non-Communicable Diseases				
None	63.1	36.9	16.6	
One	43.2	56.8	28.0	
Two or over	31.6	68.4	55.4	< 0.001
Chronic pain				
No	33.3	66.7	34.6	
Yes	43.7	56.3	65.4	0.003
ADL difficulty				
None	42.2	57.8	82.5	
One or over	30.1	69.9	17.5	< 0.001
Hypertension				
No	46.1	53.9	33.3	
Yes	37.1	62.9	66.7	0.005
Diabetes				
No	41	59	75	
Yes	37.3	62.7	25	0.262
Cardiovascular diseases				
No	40.6	59.4	77.1	
Yes	38.5	61.5	22.9	0.477
Arthritis				
No	49.6	50.4	68.1	
Yes	19.6	80.4	31.9	< 0.001
Cognitive Function				
Normal	40.7	59.3	89.6	
Low	34.3	65.7	10.4	0.179
BMI				
Normal	47.7	52.3	38.9	
Undernutrition	41.6	58.4	13.3	
Overweight	43.9	56.1	15.5	
Obesity	27.3	72.8	32.4	< 0.001

Note: ^a^ CRP and quality of life measures are presented as mean and 95% confidence interval

**Fig. 1:**
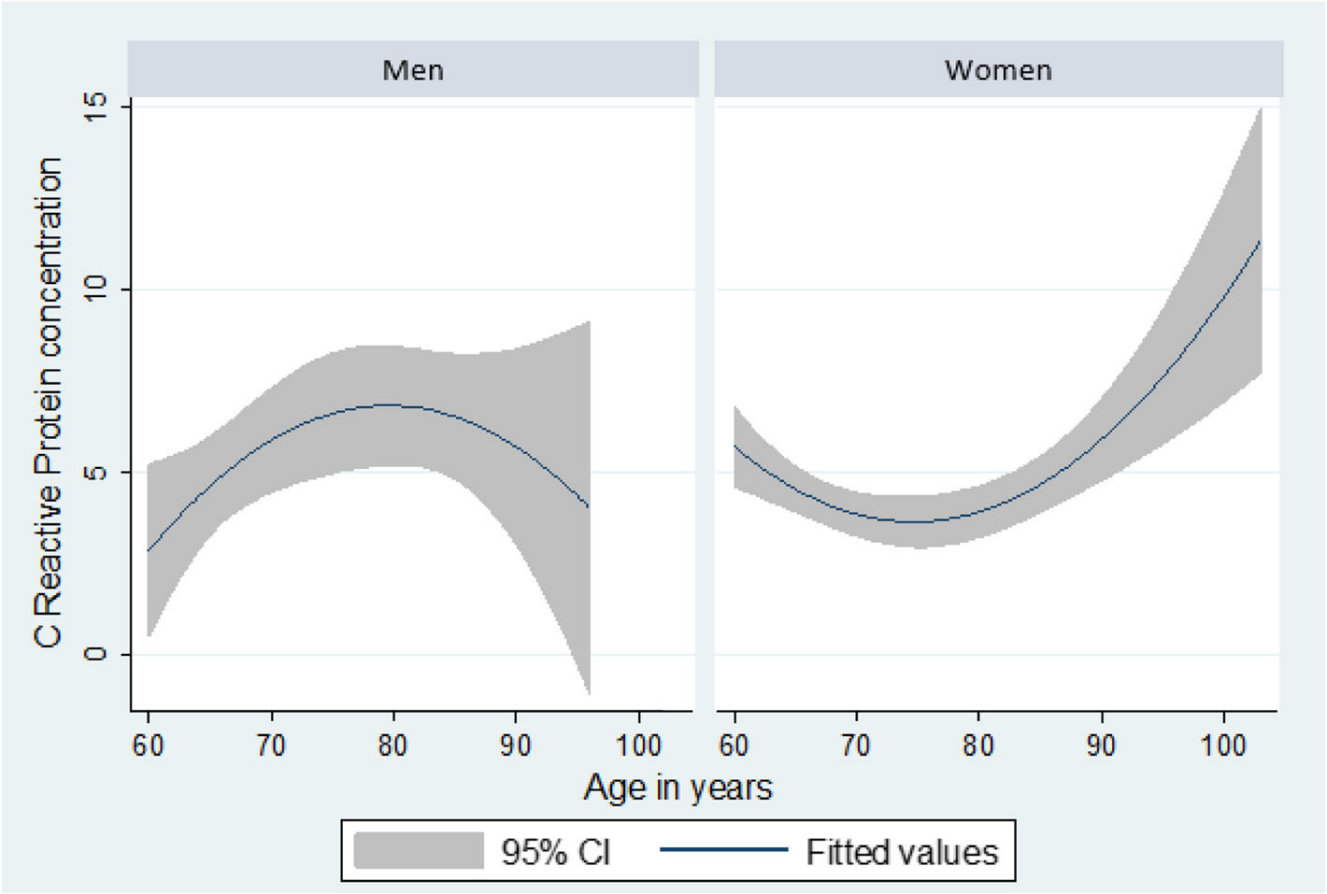
Relationship between C Reactive Protein and age, by gender. The SABE Survey (Health, Well-Being, and Aging), Brazil, 2010

Average serum levels of CRP were higher in men compared to women. However, there were some age and sex differences in the distribution of CRP serum levels. For men aged 75 and older there was a decrease in CRP serum levels, while the opposite pattern was observed among women (Fig. 1).

Higher CRP serum levels were found in participants who reported one or more chronic disease, in particular hypertension (OR 1.58, 95% CI 1.15;2.17), had a BMI value greater than 30 (OR 2.12, 95%CI 1.60;2.81), and achieved low scores in the physical domain of the SF12 (OR 1.68, 95%CI 1.28;2.21). Despite high CRP serum levels usually being associated with rheumatic conditions, we did not find an association between CRP serum levels and self-reported arthritis (Table 2). The fully adjusted model showed a strong relationship between very low scores in the physical domain of the SF12 measure (OR 1.34, 95%CI 1.02;1.77) and high BMI values (≥30) (OR 2.05, 95%CI 1.50;2.81) with CRP levels greater than 3.0 mg/L (Table 3).

**Table 2:** Unadjusted logistic regression investigating the association between elevated levels of C Reactive Protein (> 3.0 mg/L) and independent variables. The SABE Survey (Health, Well-Being, and Aging), Brazil, 2010

Variables	OR	95% CI
Socio-Demographic Conditions		
Gender		
Female	1.28	(0.97;1.69)
Age		
70–79	1.02	(0.76;1.36)
80 and more	0.85	(0.63;1.15)
Educational level		
4–7 years	0.95	(0.66;1.37)
1–3 years	1.27	(0.86;1.88)
Illiterate	1.10	(0.72;1.68)
Financial sufficiency		
No	1.17	(0.90;1.52)
Health Conditions		
Number of Non-Communicable Diseases		
One	1.29	(0.80;2.10)
Two or over	1.57	(1.04;2.38)
Chronic pain		
Yes	1.08	(0.85;1.39)
Hypertension		
Yes	1.58	(1.15;2.17)
Diabetes		
Yes	1.37	(1.01;1.86)
Cardiovascular diseases		
Yes	1.15	(0.88;1.51)
Arthritis		
Yes	1.26	(1,01;1,58)
Cognitive Function		
Compromitted	1.36	(0.98;1;89)
Difficulty in ADL		
One or over	1.32	(0.94;1.84)
BMI		
Undernutrition	0.47	(0.30;0.74)
Overweight	1.35	(0.88;2.05)
Obesity	2.12	(1.60;2.81)
Waist circumference		
with risk	0.44	(0.33;0.59)
Quality of Life Measure		
SF12 (tertiles)		
Physical domain		
Second tertile	1.31	(0.96;1.78)
Poorest tertile	1.68	(1.28;2.21)
Mental domain		
Second tertile	1.07	(0.80;1.44)
Poorest tertile	1.41	(1.04;1.92)

**Table 3:** Adjusted logistic regression investigating the association between elevated levels of C Reactive Protein (> 3.0 mg/L) and independent variables. The SABE Survey (Health, Well-Being, and Aging), Brazil, 2010

Variables analyzed	OR	95% CI
SF12 – PD^a^		
Second tertile	1.08	(0.77;1.50)
Poorest tertile	1.34	(1.02;1.77)
BMI		
Undernutrition	0.45	(0.27;0.76)
Overweight	1.25	(0.79;1.98)
Obesity	2.05	(1.50;2.81)
Gender		
Female	1.07	(0.79;1.46)
Age		
70–79	1.06	(0.73;1.54)
80 and more	0.83	(0.58;1.19)
Cognitive Function		
Compromitted	1.54	(0.99;2.39)

Note: ^a^ Physical domain of SF12 - reference: highest tercile

Regression analysis adjusted by gender, age and cognitive function

## Discussion

This study investigated the association between reduced quality of life and elevated CRP serum levels, adjusted for a wide range of covariates, in a representative sample of community-dwelling older Brazilian adults. Our main findings showed some evidence of age and gender differences in the distribution of CRP serum levels. We also found a significant association between low scores in the physical domain of the SF12 questionnaire and high CRP serum levels. Additionally, BMI values greater than 30 presented a significant relationship with high CRP serum levels.

Some authors consider aging as a progressive degenerative process highly associated with inflammation [4, 31]. However, our findings suggest that chronic inflammation demonstrates different patterns in relation to gender and increasing age, with women aged 75 and older suffering more from conditions associated with chronic inflammation. Casimir and Duchateau [32] attributed this process to hormone conditions while other studies have indicated that an increase in visceral adipose tissue may also interfere [4, 33, 34]. Although our findings may not seem expressive in absolute numbers, they reflect a common sample characteristic found in developing countries where there are more participants in the younger age group.

Reduced health-related quality of life has been associated with obesity [34]. Our analysis showed that obese older adults presented a greater reduction in the physical health domain compared to the mental well-being domain. Although the presence of co-morbidities might also be partly responsible for this observation, earlier studies have shown that a higher BMI is associated with lower HR-QoL [35, 36]. One possible explanation for this relationship is that obesity only affects mental well-being in individuals whose obesity is accompanied by binge eating or chronic diseases [36, 37].

This study also provides some evidence of the association between high CRP serum levels and low QoL levels in the physical domain. These results are consistent with another study that suggested lower scores of quality of life were associated with higher serum levels of C-reactive protein in older adults [38, 39]. The majority of evidence has shown a relationship between CRP serum levels and mental quality of life. However, our findings do not confirm this association with either cognitive function or mental quality of life. Considering that a prolonged inflammatory process is likely to be associated with cognitive decline, our relatively young sample (more than 50% aged 60–69 years) could partially explain the absence of this association [40]. It is also important to highlight that while CRP is a biomarker for the underlying inflammatory process, different cut-off points used could affect the final results and make direct comparisons between studies difficult [41, 42]. However, despite having a borderline association in the fully adjusted model, cognitive function is an important aspect when considering the inflammation process in older adults.

Our findings reinforce the importance of high physical functioning levels later in life and the association with lower levels of health-related quality of life [43]. Our hypothesis is that in the long-term, worse perception of QoL could imply chronic stress, affecting inflammatory levels in older adults. This association should be investigated longitudinally. Demographic, health, and social determinants of health characteristics are well described in the literature as factors associated with chronic inflammation. Our results show that these factors, including sex, age, number of chronic diseases, and financial sufficiency did not attenuate the associations found in this older Brazilian population [44].

We also demonstrated that obesity was associated with elevated levels of CPR. This association is corroborated by Thompson et al. [45] who found that weight gain trajectories are associated with an elevated risk of inflammation. However, another study conducted in older adults showed that the interactions of CRP with obesity were not significant [46]. The inflammatory response is complex and involves numerous cytokines, soluble cytokine receptors, acute phase reactants, and other circulating factors. In addition, circulating levels of inflammatory markers are elevated in obese individuals and correlate with BMI, total body fat, and abdominal fat in older adults. Aging related changes in body composition may also influence these different associations [47].

Our study has several strengths and potential limitations that should be acknowledged. First, this study was conducted with a large representative sample of community-dwelling older adults from São Paulo city using a wide range of covariates. Certified examiners following standardised protocols, assuring excellent quality of data, performed all examinations and laboratory measurements. Limitations arise from the cross-sectional study design, which investigates associations but cannot provide evidence of causality. Only 93.4% of the cohort participants who took part in the baseline were included in this analysis, increasing the risk of selection bias towards the inclusion of healthier participants. Another limitation could be related to the assessment of quality of life using the SF12 questionnaire, i.e., despite its good feasibility in epidemiological studies and common use in population surveys, this measure could overestimate quality of life. It is also important to highlight the lack of consensus as to which biomarker is the most accurate to evaluate inflammation. Interleukin-6 has been presented as the best biomarker for this purpose; however, it was not available in our dataset. Another point is the lack of consensus in the literature regarding the reference values for CRP, thus we opted for the cut-off point most widely used in the literature.

## Conclusion

In summary, our study suggests a significant association between lower scores in the physical domain of quality of life and high CRP serum levels, i.e., chronic inflammation in older Brazilian adults. In addition, obesity presented a significant relationship with high CRP serum levels. Our findings highlight the impact of psychosocial factors, such as quality of life, on health status, particularly non-biological mechanisms related to chronic inflammation. Future longitudinal studies are desirable to evaluate not only the relationship between quality of life and inflammation, but also to clarify the direction of these associations.

## Abbreviations

95%CI: 95% confidence interval
ADL: Activities of daily living
BMI: Body mass index
CRP: C-reactive protein
ELISA: Enzyme-linked immunosorbent assay
HR-QoL: Health Related Quality of Life
NCD: Non communicable diseases
OR: Odds ratio
QoL: Quality of life
SABE: Health, Well-being and Aging study
WC: Waist circumference
